# Vulcanite Litigation

**Published:** 1874-02

**Authors:** Samuel S. White


### ARTICLE VI.

### *Vulcanite Litigation.*

PORTLAND, January 17th, 1874.

On the fourteenth, fifteenth, and sixteenth insts., before the Hon. George F. Shepley, United States Circuit Judge, sitting in equity at Portland, Maine, the cause of the Goodyear Dental Vulcanite Company and Josiah Bacon against Daniel H. Smith was argued. This was the case selected by the Company and Mr. Bacon from those in which were set up the defences already made familiar to the readers of the *Dental Cosmos* in the supplement to the November number. Mr. Dickerson and Mr. Lee argued the complainants' case. Mr. Baldwin presented the points of defence, and in connection therewith read and filed a written argument submitted by Judge Black.

Judge Black was unable to attend in person, by reason of engagements which detained him at Washington. Dr. Smith was also represented by E. O. Shepard, Esq., of the firm of Messrs. Jewell, Gaston & Field, of Boston, his immediate counsel of record.

The argument occupied some seventeen hours, about equally divided between the complainants and the defendant.

The Judge patiently and indulgently heard the case presented at the greatest length desired by either party, and for that purpose extended the time very largely beyond the limits fixed by the rules of the court. We purpose to publish at least a synopsis of the argument on both sides, but not until the decision shall have been announced. Meanwhile, we congratulate the profession upon the fact that the hearing has been had, the questions presented and submitted, and the determination of those questions confided to a Judge whose deportment and character give an impress to the argument of the cause which assures us that, whatever may be his conclusions, they will be the fair convictions of a judicial mind after careful consideration, and with a clear comprehension of all the matters of fact and of law involved in the record.

When the decision will be announced we cannot conjecture, but it will, no doubt, be promptly rendered, and we can now only look for it with the solicitude which all the profession will share, and with the sanguine expectation based upon the same grounds which have always been our reliance, and which, after being argued faithfully, have been confided to a court which commands universal respect.

With the publication of the arguments we shall take occasion to make a somewhat extended statement of our own attitude in this litigation, such prominence having been given thereto by the complainants' counsel at the hearing as to make it a leading feature of their side of the case ; otherwise we should not feel it incumbent on us to say a word in that behalf.

SAMUEL S. WHITE.

